# Supplementary material for: Flexible and stable high-energy lithium-sulfur full batteries with only 100% oversized lithium
Source: Nat Commun. 2018 Oct 26;9:4480. doi: 10.1038/s41467-018-06879-7 (PMC6203774; doi:10.1038/s41467-018-06879-7)
Supplement: Supplementary file 1 — Supplementary Information [file 41467_2018_6879_MOESM1_ESM.pdf]

Supplementary Information

Flexible and stable high-energy lithium-sulfur full batteries with only 100% oversized lithium

Jian Chang[1], *Jian Shang*[1], *Yongming Sun*[2], *Luis K. Ono*[3], *Dongrui Wang*[1], *Zhijun Ma*[1], *Qiyao Huang*[1], *Dongdong Chen*[1], *Guoqiang Liu*[1], *Yi Cui*[2, 4], *Yabing Qi*[3], *and Zijian Zheng*[1]*

[1]Laboratory for Advanced Interfacial Materials and Devices, Institute of Textiles and Clothing, The Hong Kong Polytechnic University, Hong Kong, S. A. R., China

[2]Department of Materials Science and Engineering, Stanford University, Stanford, CA 94305, USA

[3]Energy Materials and Surface Sciences Unit, Okinawa Institute of Science and Technology Graduate University, 1919-1 Tancha, Onna-son, Okinawa 904-0495, Japan

[4]Stanford Institute for Materials and Energy Sciences, SLAC National Accelerator Laboratory, 2575 Sand Hill Road, Menlo Park, CA 94025, USA

*email: tczzheng@polyu.edu.hk

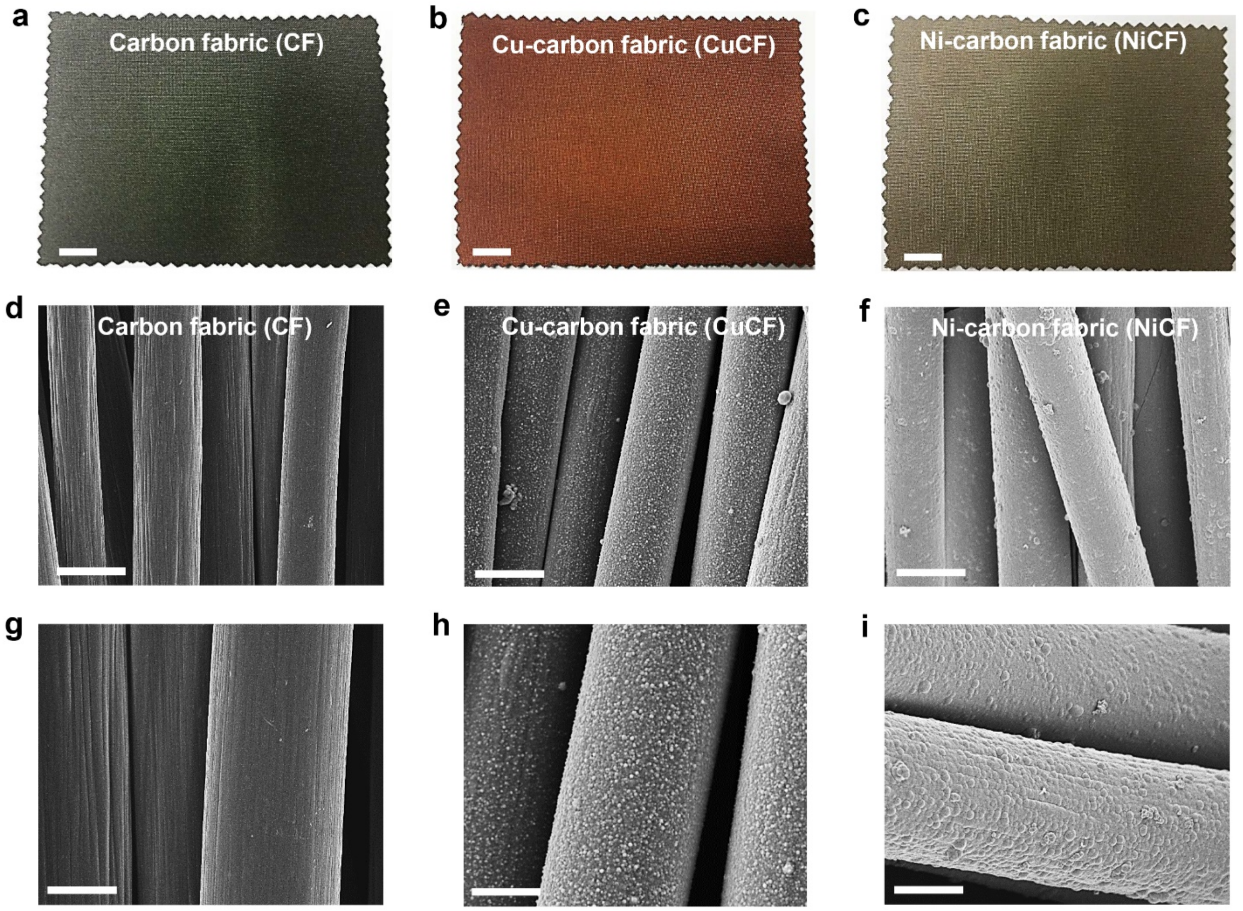

Supplementary Figure 1. Morphological characterizations of copper/nickel-coated carbon fabrics. a-c Digital images of pristine CF, CuCF, and NiCF (scale bar = 2.0 cm). **d-i** Low and high magnification SEM images of pristine CF (d, g), CuCF (e, h) and NiCF (f, i). Scale bar for d-f is 10 μm; scale bar for g-i is 5 μm.

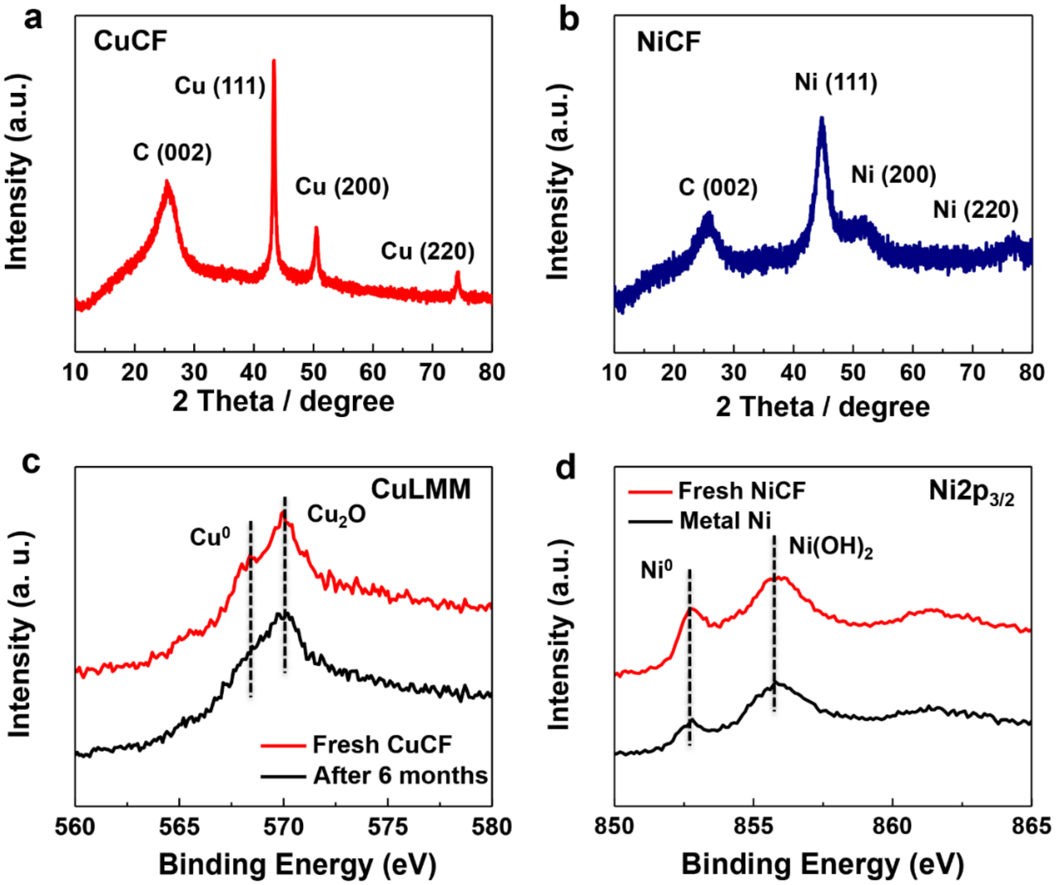

Supplementary Figure 2. Structural and compositional characterizations of copper/nickel-coated carbon fabrics. a, b X-ray diffraction patterns of CuCF and NiCF. **c** Auger Cu LMM spectroscopy of CuCF. **d** $Ni2p_{3/2}$ XPS spectroscopy of NiCF and metal Ni. The surface metal oxides of metal nanoparticles are lithiophilic, which is beneficial to the reduction of nucleation barrier and uniform Li metal deposition.

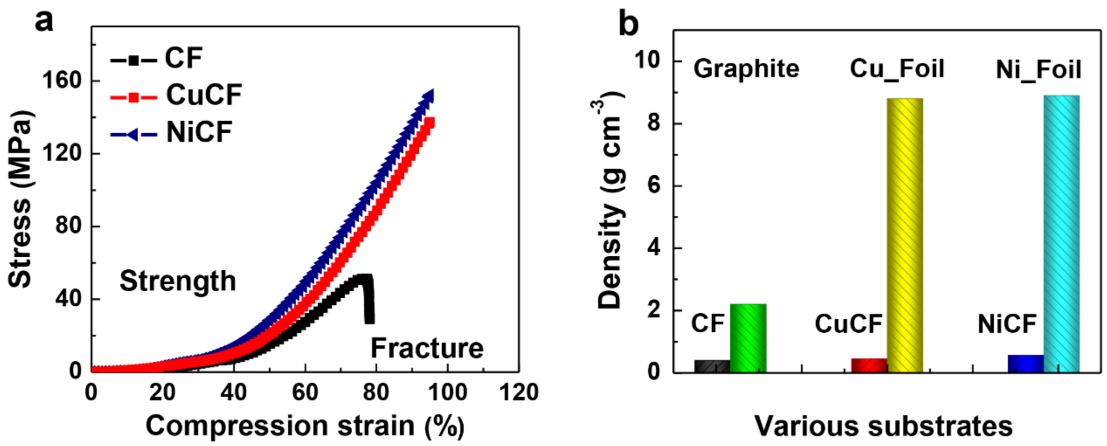

Supplementary Figure 3. Strength and density characterizations of copper/nickel-coated carbon fabrics. **a** Typical compression stress-strain curves. **b** Packing densities of graphite foil, copper foil, nickel foil, CF, CuCF and NiCF.

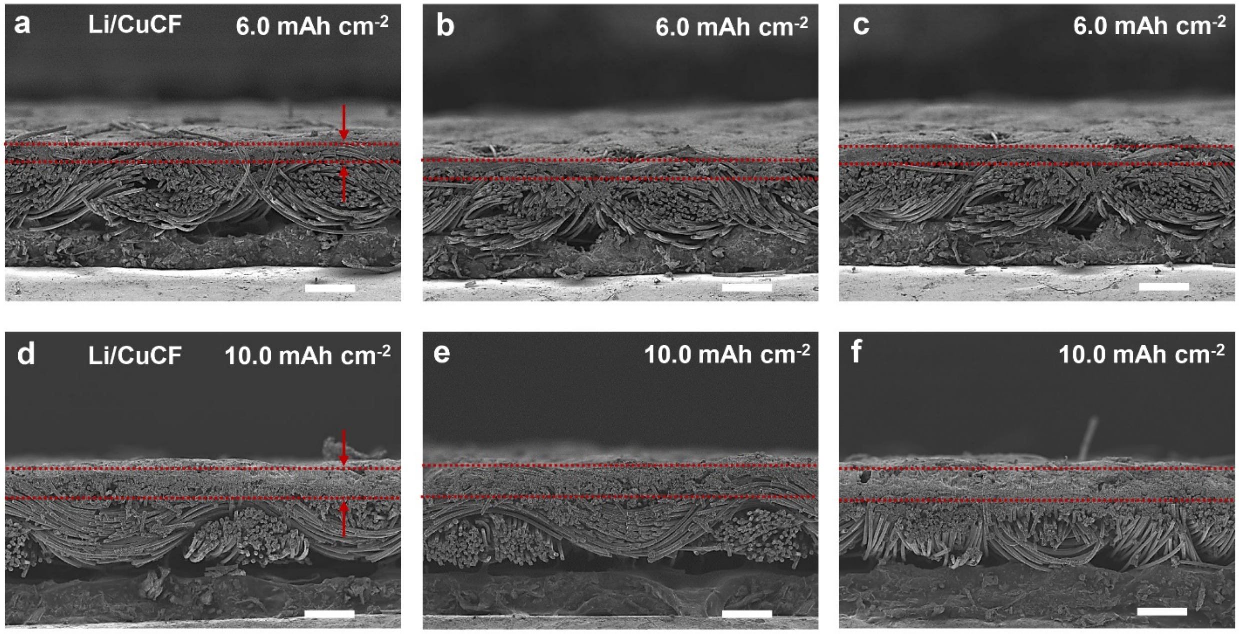

Supplementary Figure 4. Low magnification cross-sectional SEM characterizations of lithium anodes over a wide range of area. a-c SEM observations of Li/CuCF (6.0 mAh cm^{-2}) at various locations. **d-f** SEM observations of Li/CuCF (10.0 mAh cm^{-2}) at various locations. The Li metal prefers to deposit on the top part of CuCF due to the shorter Li^+ diffusion pathway compared

to the bottom part. With increasing the areal capacity of Li/CuCF anode, the deposition of Li metal gradually expands from the top to the bottom of CuCF. Scale bar, 100 μm.

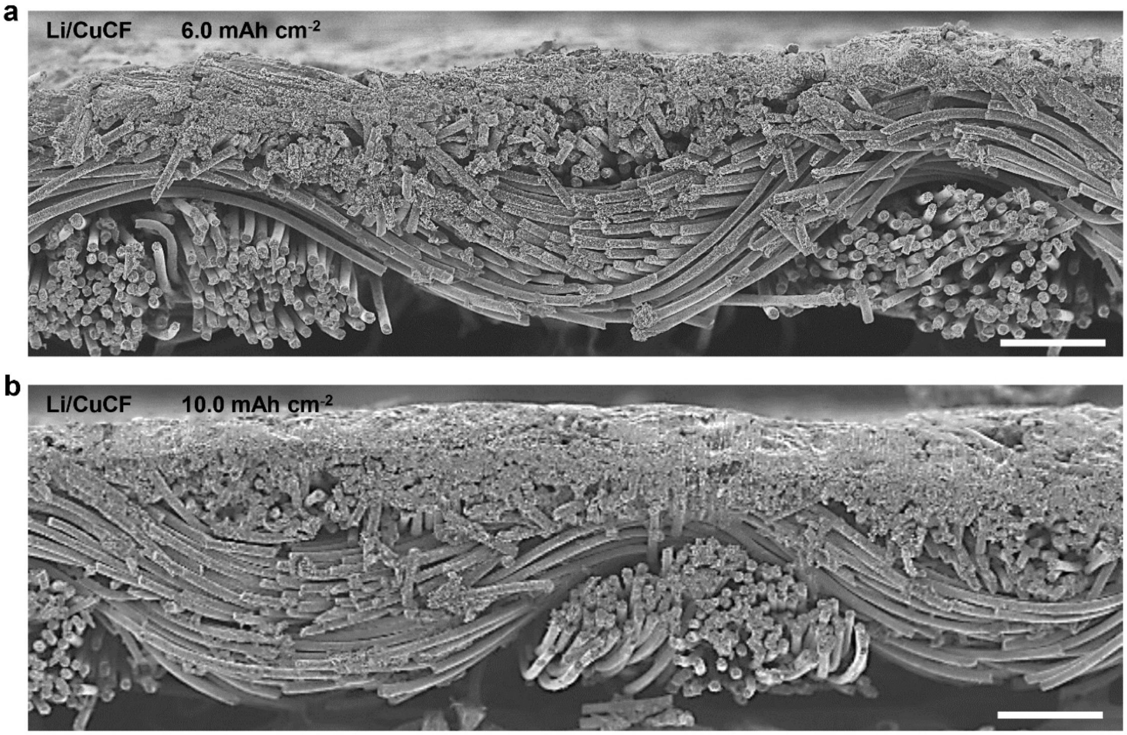

Supplementary Figure 5. Enlarged SEM characterizations of lithium anodes with various areal capacities in the through-thickness direction. a 6.0 mAh cm^{-2}. **b** 10.0 mAh cm^{-2}. Scale bar, 100 μm.

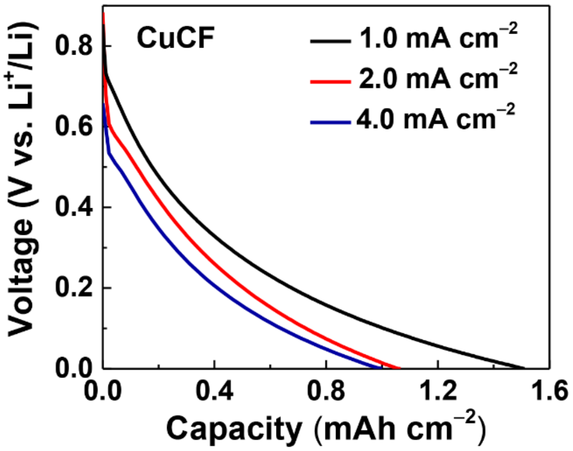

Supplementary Figure 6. The partial lithiation for lithium anodes can be regulated with changing current densities during electrodeposition.

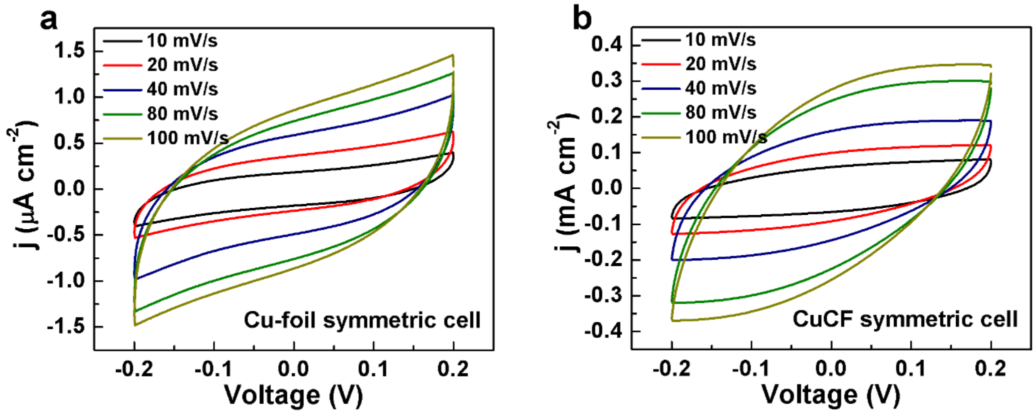

Supplementary Figure 7. The surface area characterizations of copper-coated carbon fabrics. a, b Cyclic voltammetry curves of symmetric cells based on bare Cu foil and CuCF. Here, the current density of CuCF is an order of magnitude higher than that of Cu foil, indicating accessible electrochemical surface area of CuCF is much higher than that of Cu foil.

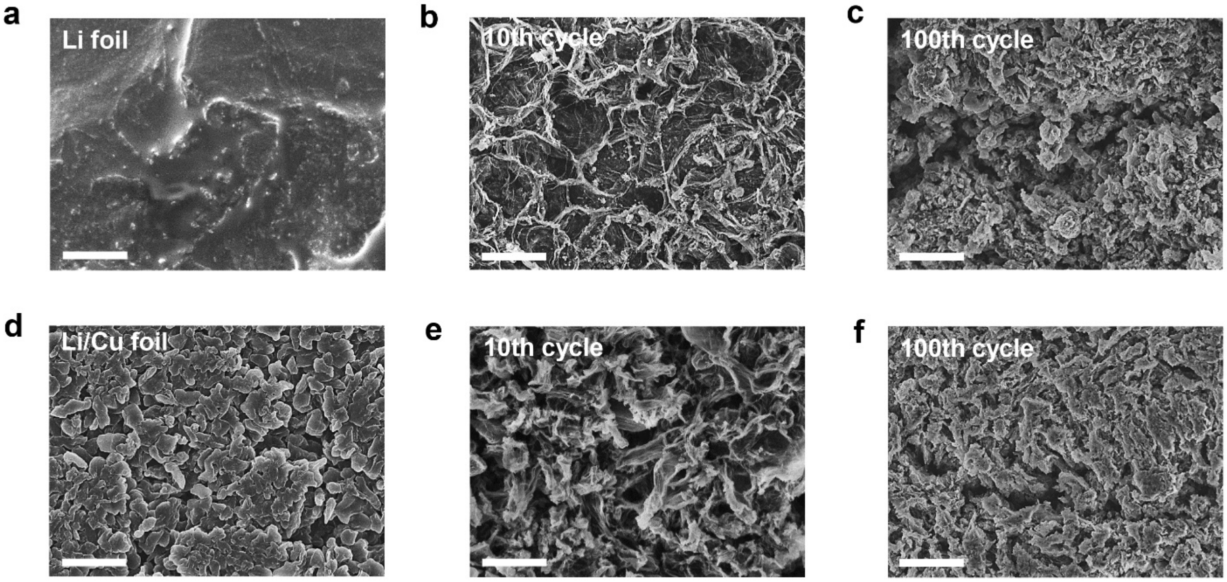

Supplementary Figure 8. Morphological characterizations of lithium anodes after cycling. a-c SEM images of bare Li foil at the 1st cycle, after the 10th cycle and after the 100 cycles. **d-f** SEM images of bare Cu foil at the 1st cycle, after the 10th cycle and after the 100 cycles. The amount of Li cycled is 2.0 mAh cm^{-2} at 1.0 mAcm^{-2} in all cases. Scale bar, 5 µm.

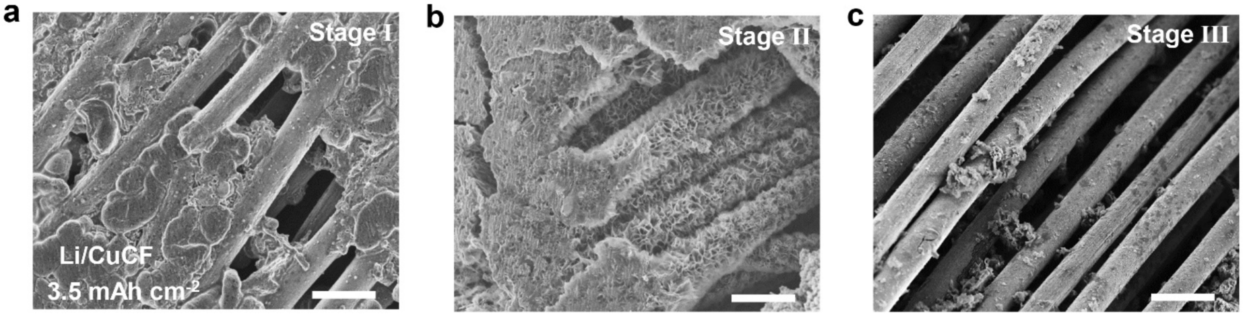

Supplementary Figure 9. Morphological evolution of lithium anodes in three cycling Stages (I, II, and III). a SEM images of Li/CuCF anode with uniform spherical particles of Li metal in State I. **b** SEM images of Li/CuCF anode with well-formed Li nanoflakes in Stage II. **c** SEM images of Li/CuCF anode without Li metal residues in Stage III. Scale bar, 20 µm.

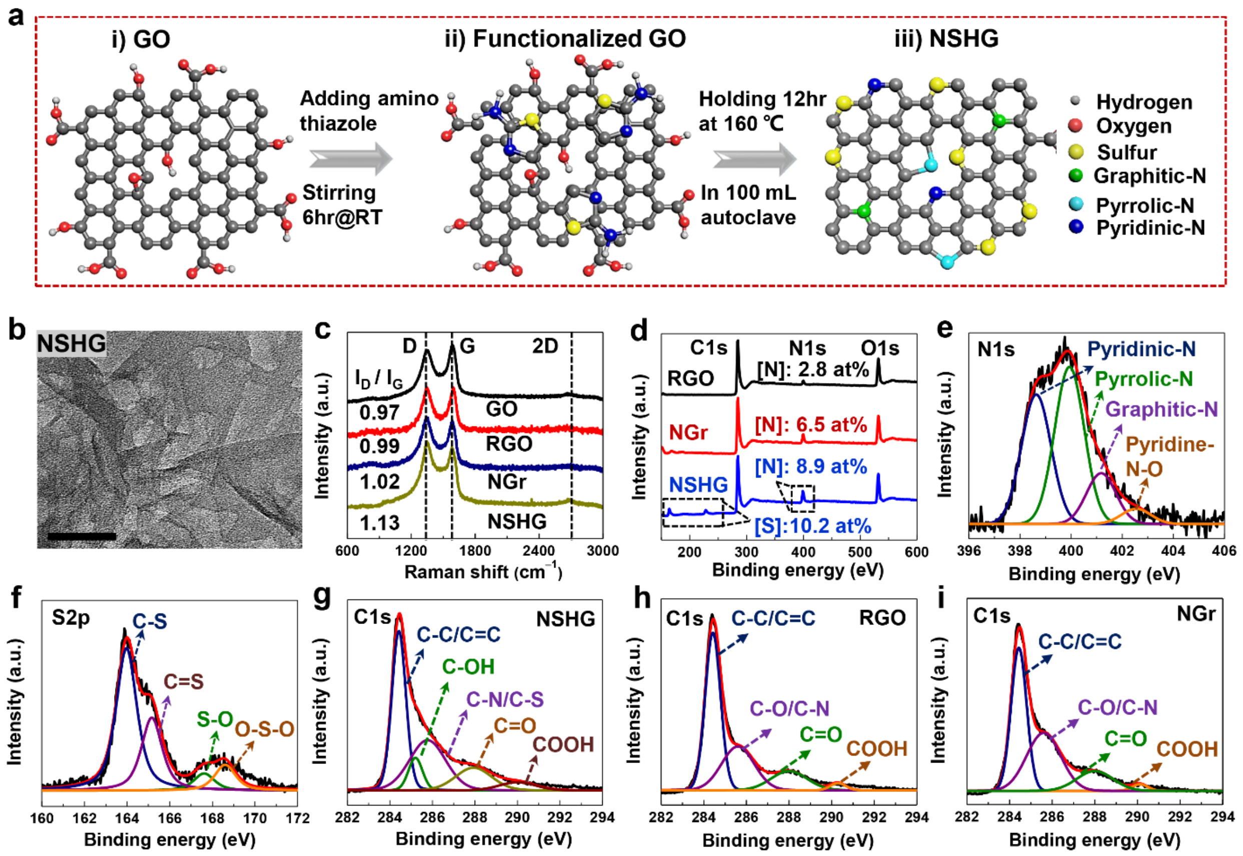

Supplementary Figure 10. Synthesis and characterizations of nitrogen and sulfur heavily doped graphene serving as polar binders. a Schematic illustration of synthesizing NSHG by a facile hydrothermal method. **b** High-magnification TEM images of NSHG with porous structures. Scale bar, 50 nm. **c** Raman spectroscopy of GO, RGO, NGr, and NSHG. The high intensity ratio of D to G band (1.13) of NSHG suggests the doping of nitrogen and sulfur into graphene. **d** X-ray photon spectroscopy (XPS) survey spectra of RGO, NGr and NSHG. **e, f** High-resolution N_{1s} and S_{2p} XPS core spectra of NSHG. **g-i** High-resolution C_{1s} XPS core spectra of NSHG, RGO, and NGr. Here, high-resolution N1s XPS spectra of NSHG indicate the existence of pyridinic N (398.7 eV), pyrrolic N (400.1 eV), and graphitic N (401.2 eV) structures. Moreover, the deconvoluted spectra of S2p suggests the binding states of sulfur are C-S-C (163.9 eV), C=S (165.2 eV), S-O (167.6 eV) and O-S-O (168.6 eV). High-resolution C1s XPS spectra of NSHG reveals small amount of oxygen-containing functional groups at 285.2 (C-OH), 288 (C=O), and 290 (O=C-O) are retained.

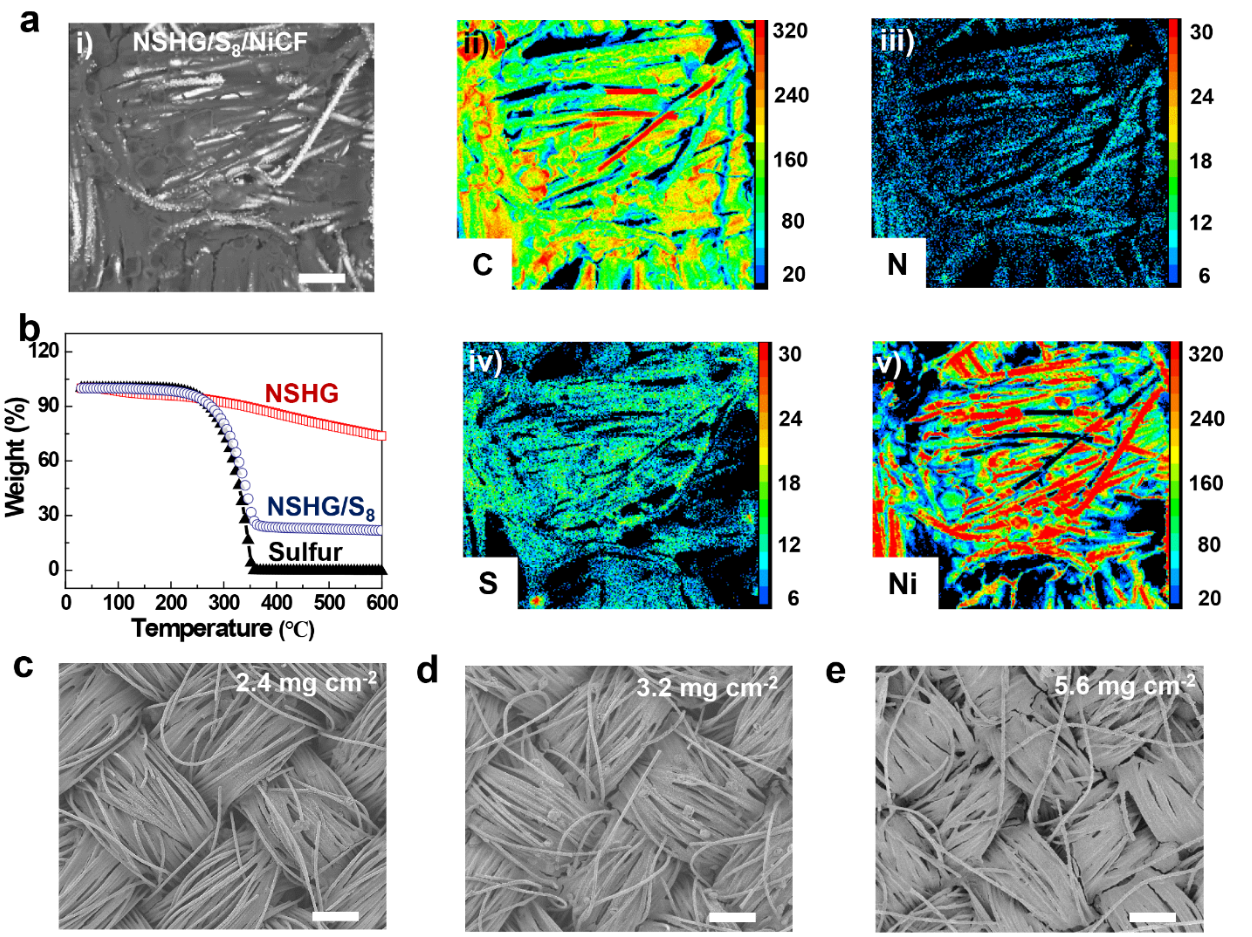

Supplementary Figure 11. Compositional and morphological characterizations of sulfur cathodes. a Elemental (C, N, S, and Ni) distribution mapping and their corresponding quantitative composition in the composite cathode ($NSHG/S_8/NiCF$) precisely detected by electron probe micro-analyzer (EPMA). Scale bar, 50 μm. **b** Thermalgravimetric analysis (TGA) of sulfur, NSHG, and $NSHG/S_8$ (containing Super P). **c-e** SEM images of cathodic $NSHG/S_8/NiCF$ with different mass loadings of 2.4 mg cm^{-2}, 3.2 mg cm^{-2}, and 5.6 mg cm^{-2}, respectively. Scale bar, 100 μm.

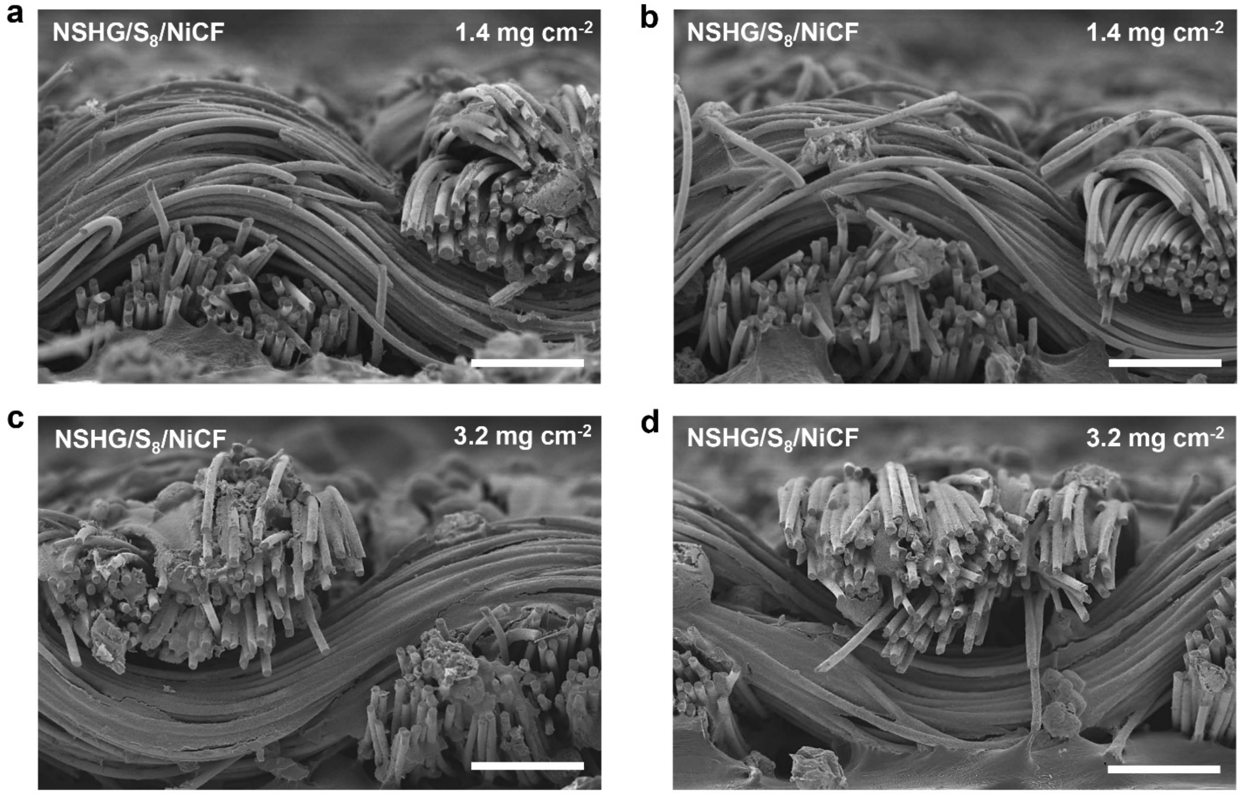

Supplementary Figure 12. Cross-sectional SEM characterizations of sulfur cathodes at various locations. a, b SEM images of NSHG/S_8/NiCF with a sulfur loading of 1.4 mg cm^{-2}. **c, d** SEM images of NSHG/S_8/NiCF with a sulfur loading of 3.2 mg cm^{-2}. Scale bar, 100 µm.

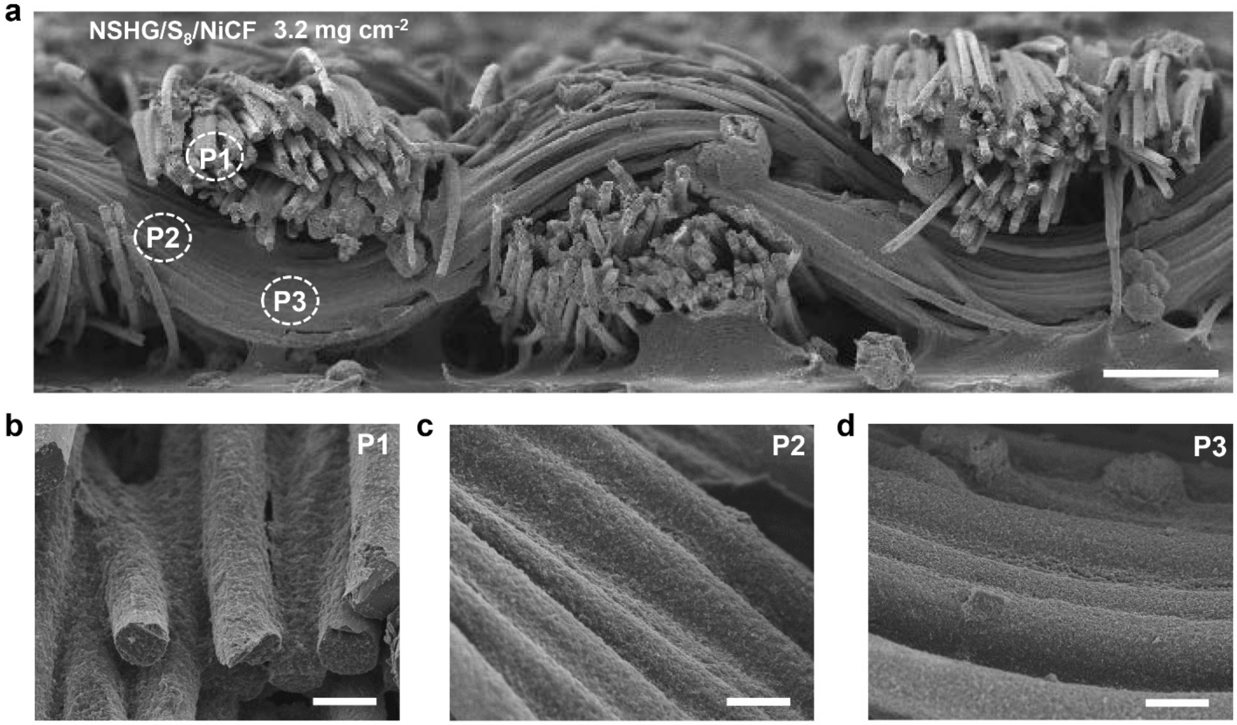

Supplementary Figure 13. Cross-sectional SEM characterizations of sulfur cathodes in the through-thickness direction. a Low magnification SEM images of NSHG/S_8/NiCF with a wide range of cross-sectional area. Scale bar, 100 µm. **b-d** High magnification SEM images of NSHG/S_8/NiCF from top to bottom. Scale bar, 10 µm.

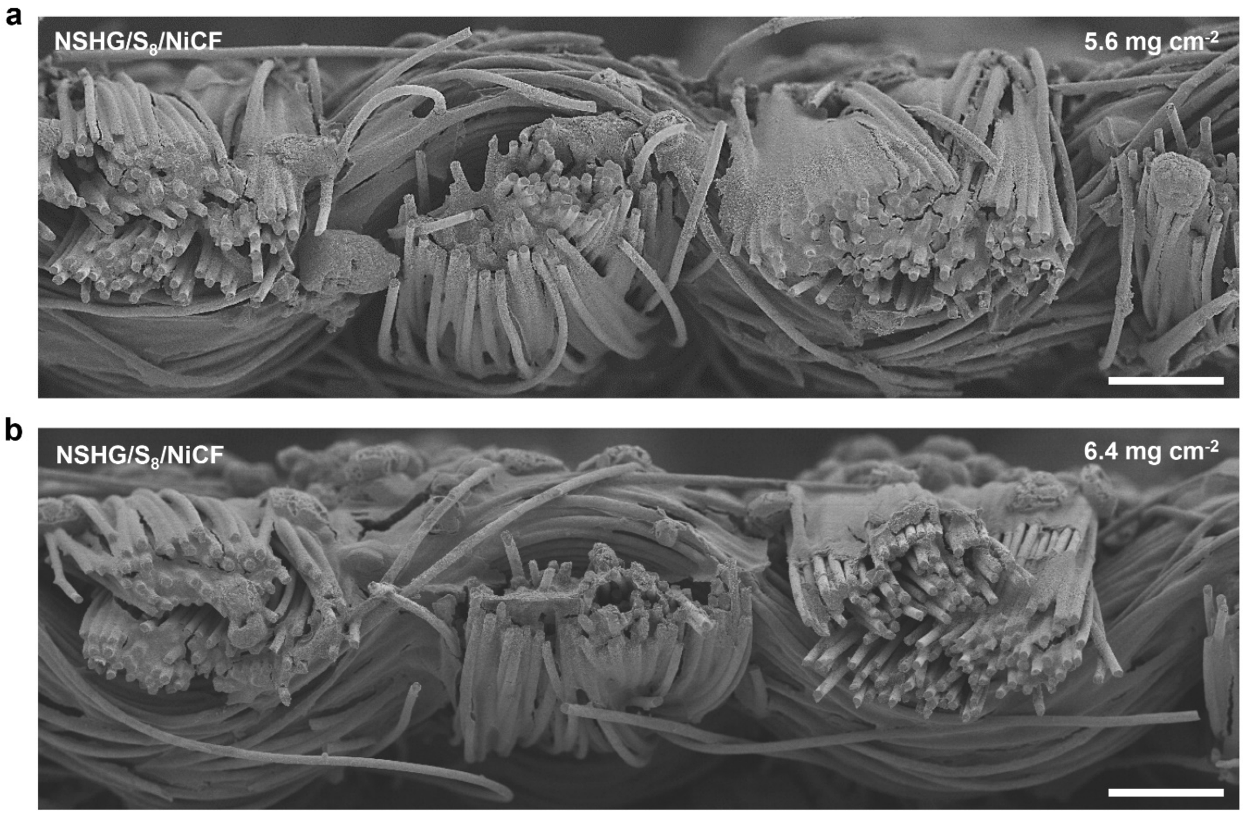

Supplementary Figure 14. Enlarged SEM characterizations of sulfur cathodes with various sulfur loadings in the through-thickness direction. a 5.6 mg cm^{-2}. **b** 6.4 mg cm^{-2}. Scale bar, 100 μm.

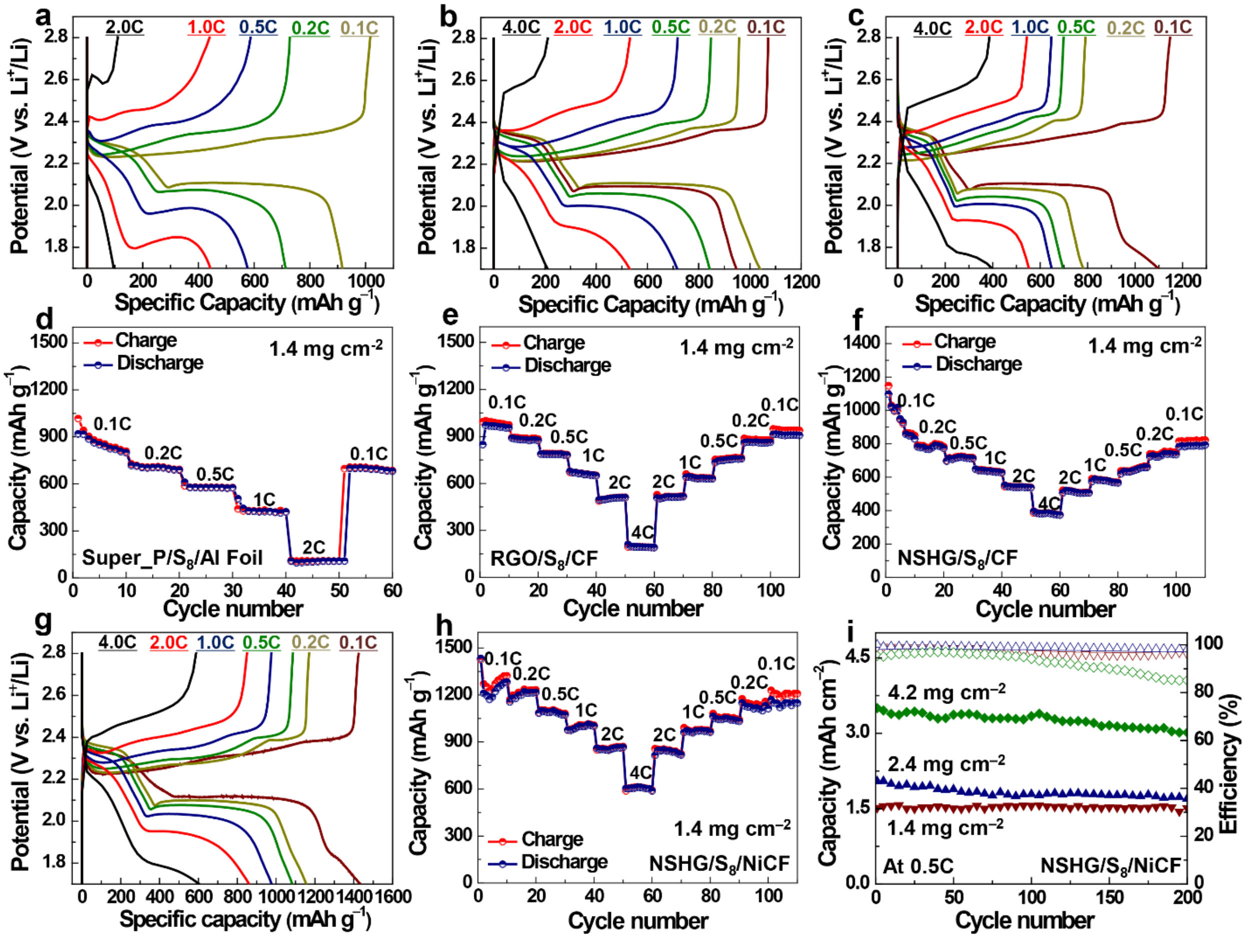

Supplementary Figure 15. Rate and cycling performance of sulfur cathodes in the half-cell. **a-h** Galvanostatic charge-discharge profiles and corresponding capacities at various rates (1 *C* = 1675 mA g^{-1} of sulfur) of Super P/S_8/Al foil (a, d), RGO/S_8/CF (b, e), NSHG/S_8/CF (c, f), and NSHG/S_8/NiCF (g, h) in half-cell. **i** Cycling performance and corresponding CEs of NSHG/S_8/NiCF were plotted with different sulfur loadings at 0.5 *C*.

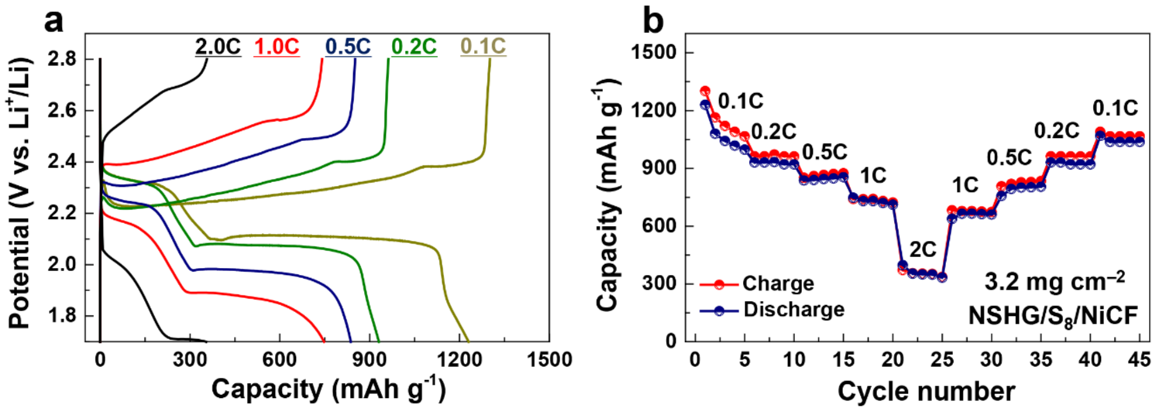

Supplementary Figure 16. Rate capability of sulfur cathode. a Galvanostatic charge-discharge profiles of NSHG/S_8/NiCF cathode (vs. Li foil) at a wide range of charge/discharge rates ranging from 0.1 C to 2 C (1 *C* = 1675 mA g^{-1} of sulfur; sulfur mass loadings: 3.2 mg cm^{-2}). **b** Specific capacities of NSHG/S_8/NiCF cathode (vs. Li foil) at different rates using the same sulfur loading of 3.2 mg cm^{-2}.

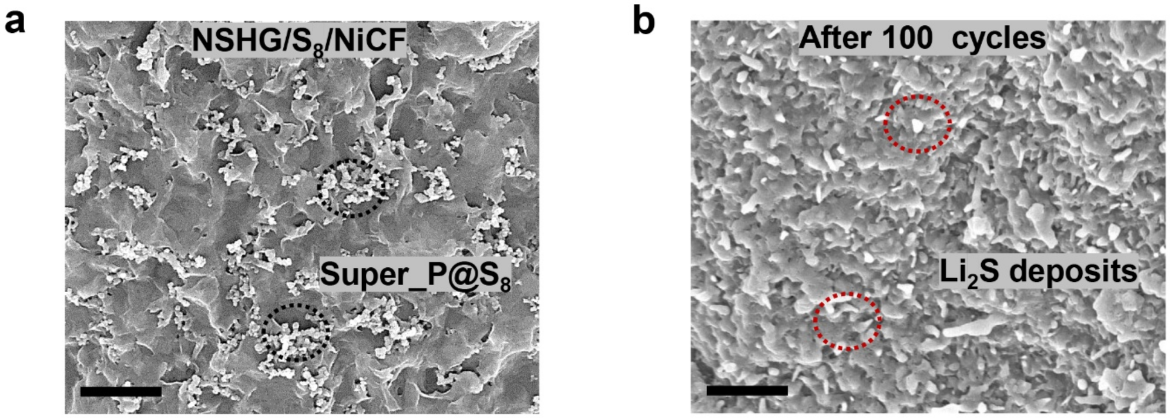

Supplementary Figure 17. **Morphological evolution of sulfur cathodes after cycling. a** SEM images of NSHG/S_8/NiCF. **b** SEM images of NSHG/S_8/NiCF after 100 charge/discharge cycles at 0.5 C. After cycling, it is observed that NiCF supports the uniform agglomerates of Li_2S nanoparticles within NSHG layers, which indicates the rapid growth of Li_2S deposits by heterogeneous nucleation onto the surface of NiCF. The strong structure could endow excellent mechanical robustness and electrochemical performance. Scale bar, 1 μm.

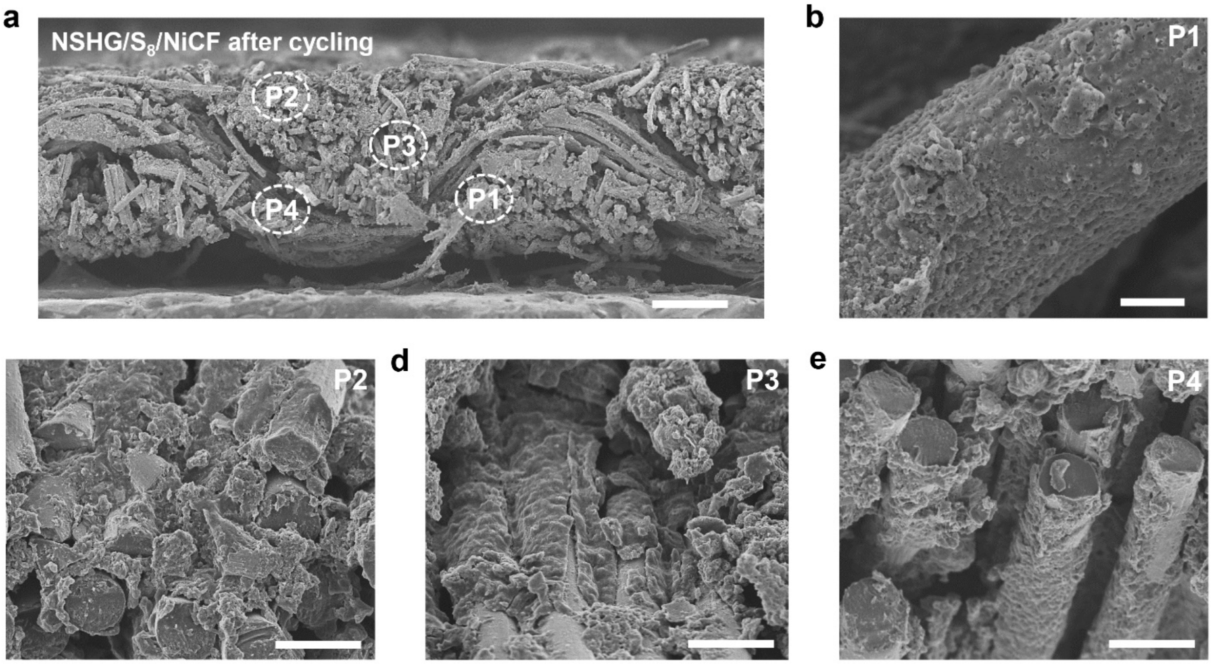

Supplementary Figure 18. **Cross-sectional SEM characterizations of sulfur cathodes after cycling in the through-thickness direction. a** Low magnification SEM images of NSHG/S_8/NiCF with a wide range of cross-sectional area. Scale bar, 100 μm. **b-e** High magnification SEM images of NSHG/S_8/NiCF from top to bottom (P1-P4). Scale bar for b is 3 μm; scale bar for c-e is 10 μm.

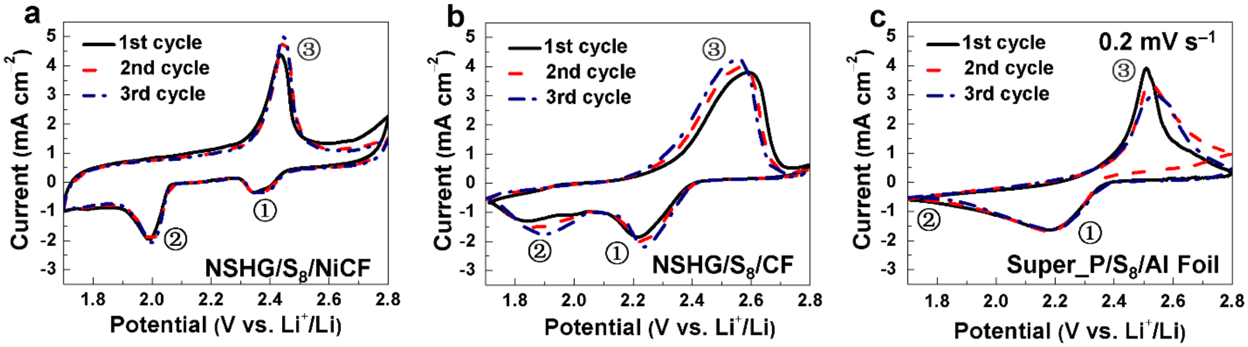

Supplementary Figure 19. **The catalytical role of nickel metal layer in sulfur cathodes. a-c** Typical cyclic voltammetry curves of NSHG/S_8/NiCF (a), NSHG/S_8/CF (b), and Super P/S_8/Al (c) electrodes within a potential window of 1.7-2.8 V at a scan rate of 0.2 mV s^{-1}.

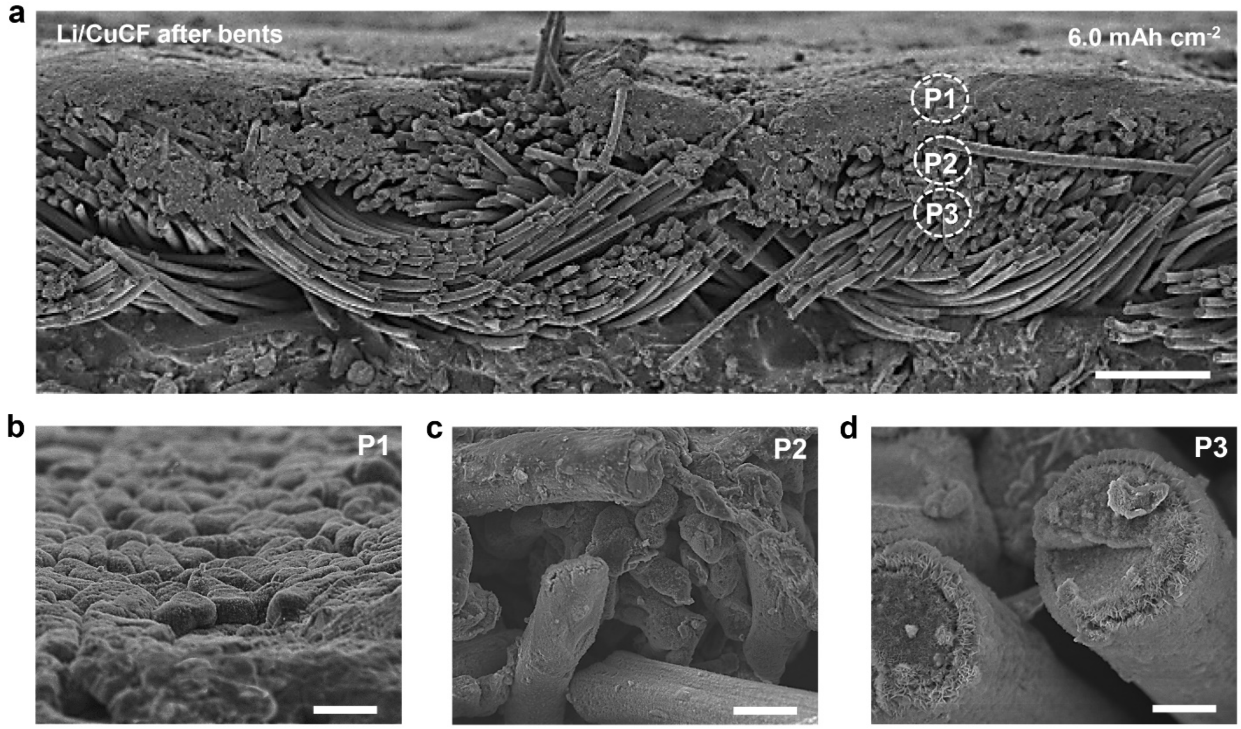

Supplementary Figure 20. **Morphological characterizations of lithium anodes after repeatable bents in the through-thickness direction. a** Low magnification SEM images of Li/CuCF with a wide range of cross-sectional area. Scale bar, 100 μm. **b-d** High magnification SEM images of Li/CuCF from top to bottom (P1-P3). Scale bar, 10 μm.

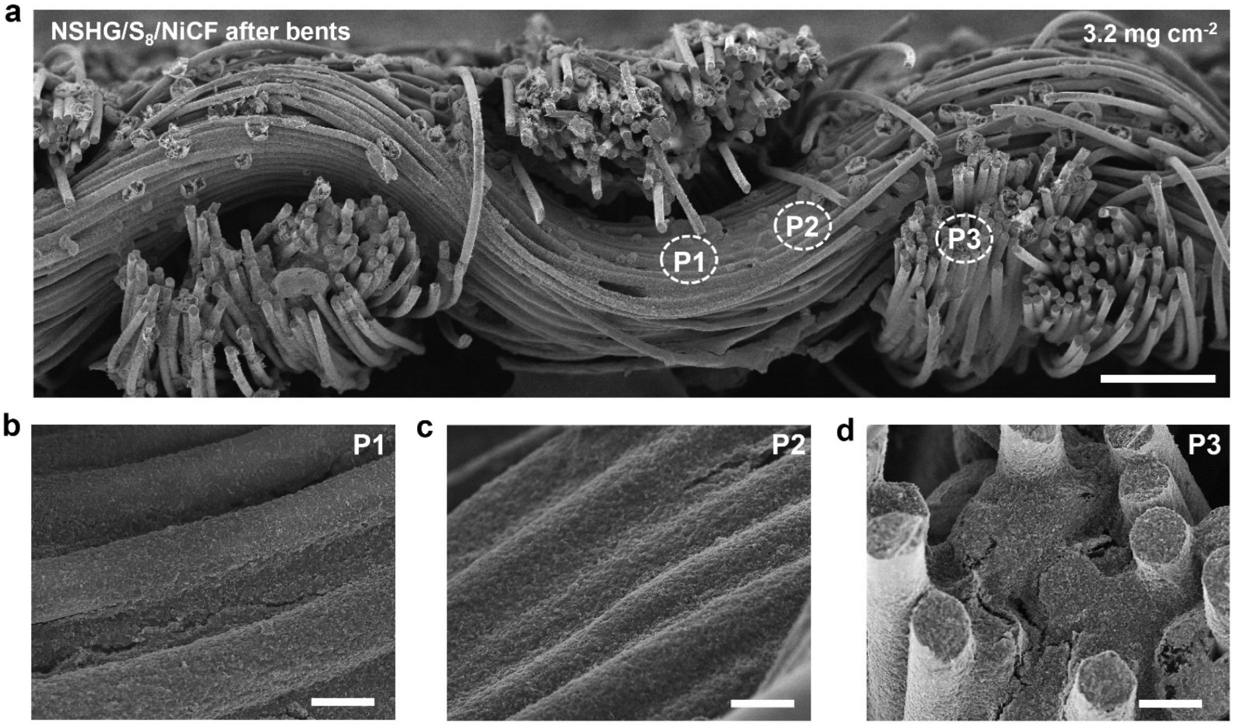

Supplementary Figure 21. **Morphological characterizations of sulfur cathodes after repeatable bents in the through-thickness direction. a** Low magnification SEM images of the NSHG/S_8/NiCF cathode with a wide range of cross-sectional area. Scale bar, 100 µm. **b-d** High magnification SEM images of the NSHG/S_8/NiCF cathode from top to bottom (P1-P3). Scale bar, 10 µm.

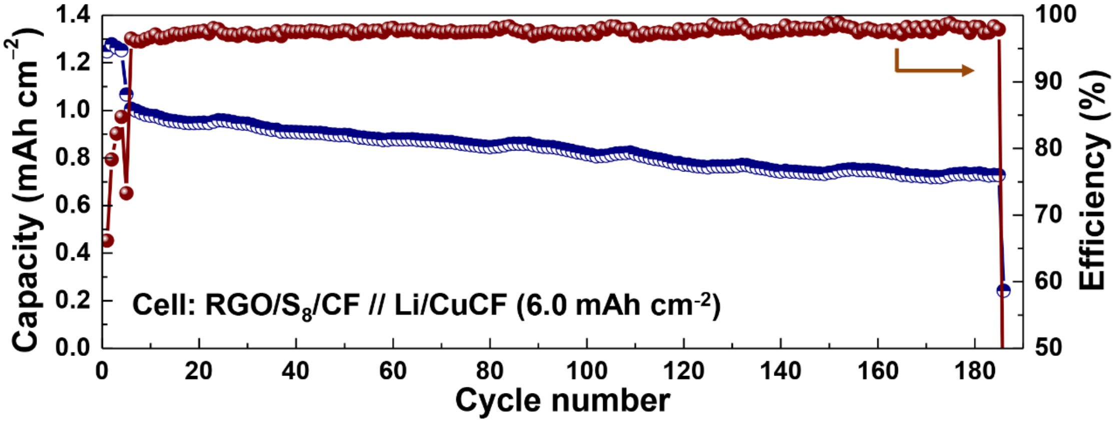

Supplementary Figure 22. The discharge capacity retention and corresponding Coulombic efficiency of full-cell. Without Ni-catalytical layers, the sulfur cathode of RGO/S_8/CF with low Coulombic efficiency leads to the overall performance decay of Li-S full cells during cycling.

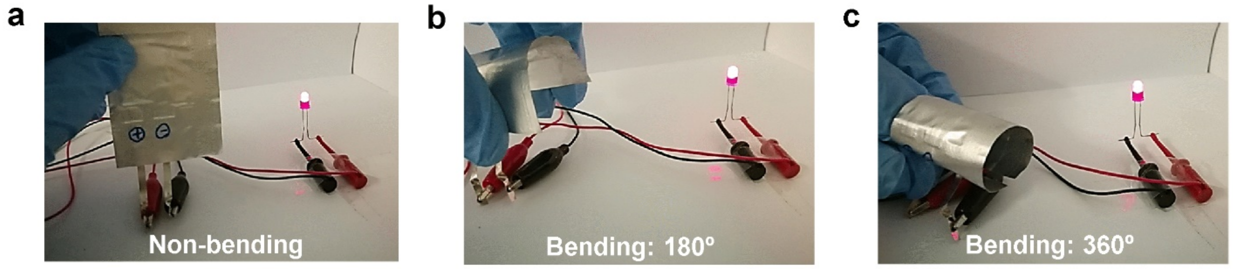

Supplementary Figure 23. The unit cell (2 ×2 cm^2) of lithium-sulfur full batteries is fabricated for powering a LED (1.5 V) under various mechanical bending conditions. a Non-bending. **b** 180° bending. **c** 360° bending.

Supplementary Table 1. Typical comparison of atomic concentration between various polar binders (RGO, NGr, and NSHG).

Atomic concentration	[C at%]	[O at%]	[N at%]	[S at%]
RGO	83.8 %	13.4 %	2.8 %	NA
NGr	80.3 %	13.2 %	6.5 %	NA
NSHG	69.1 %	11.8 %	8.9 %	10.2 %

Notably, the achieved RGO with certain N dopant originates from the reductant of ammonia.

Supplementary Table 2. Calculated surface energy of Ni thin slabs with different crystal orientations and layers.

Model of metal Ni	Ni atomic layers	Total energy (eV)	Surface energy (eV)
Bulk Ni	∞	–21.86	NA
	4	–82.29	0.119
Ni (111) slabs	7	–148.00	0.116
	10	–213. 54	0.116
Ni (100) slabs	3	-19.37	1.083
	5	-51.20	0.280
	7	-73.11	0.277
Ni (110) slabs	5	-49.67	0.286
	7	-71.48	0.289
	9	-93.35	0.289

Noted, the surface energy of the thin slabs is converged when the surface energy difference between neighboring layers is less than 0.003 eV. Therefore, the thin slab of 7 layered Ni (111) with the smallest surface energy is selected for modelling Ni nanoparticles in NiCF.

Supplementary Table 3. Performance comparison of our work and current state-of-the-art flexible lithium ion batteries (capable of multiple bending at the curvature of <30 mm).

Current Collectors	Anode	Cathode	Cell mass (mg cm^{-2})	Cell volume (cm^3)	Areal energy (mWh cm^{-2})	Energy density (Wh kg^{-1})	Energy density (Wh L^{-1})	Cell cycling number	Ref.
**CuCF, NiCF**	**Li/CuCF**	**NSHG/S_8/NiCF**	**21.88**	**0.0175**	**6.3 (2.1 V)**	**288@1.0 mA cm^{-2}**	**360**	**>80% @ 260**	**Ours**
CNT film	$Li_4Ti_5O_{12}$	$LiCoO_2$	15.00	0.0164	1.61 (2.3 V)	107@0.1 mA cm^{-2}	98	20	1
Graphene (Gr)	Lithiated-Gr	V_2O_5	0.22	0.0030	0.03 (2.1 V)	136@0.1 mA cm^{-2}	10	30	2
Carbon fabrics	$Li_4Ti_5O_{12}$	$LiFePO_4$	8.33	0.0094	0.95 (1.9 V)	114@0.16 mA cm^{-2}	101	35	3
Cu/Al foils	$Li_4Ti_5O_{12}$	$LiFePO_4$	NA	0.0169	1.69 (1.8 V)	NA	100	NA	4
Cu/Al foils	Graphite	$LiNiCoAlO_2$	12.54	NA	1.48 (3.7 V)	118@0.08 mA cm^{-2}	NA	50	5
Cu/Al tabs	$Li_4Ti_5O_{12}$	$LiCoO_2$	31.63	0.0330	2.31(2.4 V)	92@0.15 mA cm^{-2}	70	150	6
CNT sheets	$Li_4Ti_5O_{12}$	$LiMn_2O_4$	21.11	0.0317	0.57 (2.6 V)	27@0.01 mA cm^{-2}	18	100	7
Cu/Al foils	Li foil	$LiCoO_2$	NA	NA	0.40 (4.0 V)	NA	NA	100	8
Activated cotton	Li foil	NiS_2-Gr	32.84	NA	3.58 (1.4 V)	109@0.1 mA cm^{-2}	NA	200	9

Noted, the cell energy density of some flexible lithium ion batteries is calculated according to the related information (total mass/volume of current collectors, the electrodes and separator) in the references.

Supplementary Table 4. Performance of flexible lithium-sulfur full batteries.

Anode	Cathode	Li excess amount	Cell mass (mg cm^{-2})	Cell volume (cm^3)	Energy density (Wh kg^{-1})	Energy density (Wh L^{-1})	Bending cycles	Cycle life without bending	Cycle life after bending	Ref.
**Li/CuCF**	**NSHG/S_8/NiCF**	**100%**	**21.88**	**0.0175**	**288@1.0 mA cm^{-2}**	**360**	**200@ r=5 mm**	**>80% @260**	**>90% @150**	**Ours**
Li foil	PVDF/CB/S_8/Gr@PP	6813%	30.3	0.0595	99@1.1 mA cm^{-2}	50	1@ θ=90°	500	30	10
Li foil	PEDOT/S_8/Gr/Al	3042%	37.7	0.0575	183@3.3 mA cm^{-2}	120	1@ θ=180°	500	80	11
Li foil	PVDF/MOFs/S_8/CNT	2757%	33.5	0.0557	220@1.5 mAcm^{-2}	132	1@ θ=180°	200	60	12
Li foil	Gr/S_8/cotton	2594%	35.5	NA	227@1.2 mA cm^{-2}	NA	1@ θ=180°	200	50	13
Li foil	CMK-3/S_8/CNT	8542%	34.5	NA	89@0.17 mA cm^{-2}	NA	1@ θ=180°	100	50	14
Li foil	Gr/S_8/Al	6381%	31.8	0.0625	75@0.7 mA cm^{-2}	38.4	1@ θ=180°	100	20	15
Li foil	Gr nanotube/S_8	8763%	28.7	NA	86@0.17 mA cm^{-2}	NA	1@ r=10 mm	500	60	16
Li foil/CNT	PVDF/S_8/CNT	3234%	33.9	0.0568	194@2.85 mA cm^{-2}	115	100@ θ=180°	200	3	17
Li foil	Li_2S_6/carbon cloth	1974%	46.6	NA	225@1.0 mA cm^{-2}	NA	300@ r=5 mm	100	10	18

Noted, the cell energy density of previously reported Li-S batteries is calculated according to the related information (total mass/volume of current collectors, the electrodes and separator) in the references. The lithium metal foil of hundreds of micrometres thick (e.g., 500 µm) is often used in the research cell due to its very low Coulombic efficiency, ready availability and low cost from laboratory supply companies. Control parameters for bending tests include bending angle of θ and bending radius of r.

Supplementary References

1. Hu, L., Wu, H., La Mantia, F., Yang, Y. & Cui, Y. Thin, flexible secondary Li-ion paper batteries. *ACS Nano* **4**, 5843-5848 (2010).
2. Gwon, H. et al. Flexible energy storage devices based on graphene paper. *Energy Environ. Sci.* **4**, 1277-1283 (2011).
3. Choi, K.H. et al. Heterolayered, one-dimensional nanobuilding block mat batteries. *Nano Lett.* **14**, 5677-5686 (2014).
4. Kim, S.H. et al. Printable Solid-State Lithium-Ion Batteries: A New Route toward Shape-Conformable Power Sources with Aesthetic Versatility for Flexible Electronics. *Nano Lett.* **15**, 5168-5177 (2015).
5. Kim, J.S. et al. A half millimeter thick coplanar flexible battery with wireless recharging capability. *Nano Lett.* **15**, 2350-2357 (2015).
6. Song, Z. et al. Origami lithium-ion batteries. *Nat. Commun.* **5**, 3140 (2014).
7. Ren, J. et al. Elastic and wearable wire-shaped lithium-ion battery with high electrochemical performance. *Angew. Chem. Int. Ed. Engl.* **53**, 7864-7869 (2014).
8. Koo, M. et al. Bendable inorganic thin-film battery for fully flexible electronic systems. *Nano Lett.* **12**, 4810-4816 (2012).
9. Gao, Z., Song, N., Zhang, Y. & Li, X. Cotton-Textile-Enabled, Flexible Lithium-Ion Batteries with Enhanced Capacity and Extended Lifespan. *Nano Lett.* **15**, 8194-8203 (2015).
10. Zhou, G. et al. A flexible sulfur-graphene-polypropylene separator integrated electrode for advanced Li-S batteries. *Adv. Mater.* **27**, 641-647 (2015).
11. Xiao, P., Bu, F., Yang, G., Zhang, Y. & Xu, Y. Integration of Graphene, Nano Sulfur, and Conducting Polymer into Compact, Flexible Lithium-Sulfur Battery Cathodes with Ultrahigh Volumetric Capacity and Superior Cycling Stability for Foldable Devices. *Adv. Mater.* **29**, 1703324 (2017).
12. Mao, Y. et al. Foldable interpenetrated metal-organic frameworks/carbon nanotubes thin film for lithium-sulfur batteries. *Nat. Commun.* **8**, 14628 (2017).
13. Gao, Z., Zhang, Y., Song, N. & Li, X. Towards flexible lithium-sulfur battery from natural cotton textile. *Electrochim. Acta* **246**, 507-516 (2017).
14. Sun, Q. et al. An Aligned and Laminated Nanostructured Carbon Hybrid Cathode for High-Performance Lithium-Sulfur Batteries. *Angew. Chem. Int. Ed. Engl.* **54**, 10539-10544 (2015).

15. Wu, C., Fu, L., Maier, J. & Yu, Y. Free-standing graphene-based porous carbon films with three-dimensional hierarchical architecture for advanced flexible Li–sulfur batteries. *J. Mater. Chem. A* **3**, 9438-9445 (2015).
16. Chen, K. et al. Sulfur nanoparticles encapsulated in reduced graphene oxide nanotubes for flexible lithium-sulfur batteries. *Nano Res.* **11**, 1345-1357 (2018).
17. Li, L. et al. A Foldable Lithium-Sulfur Battery. *ACS Nano* **9**, 11342-11350 (2015).
18. Song, J.Y. et al. A Polysulfide-Infiltrated Carbon Cloth Cathode for High-Performance Flexible Lithium-Sulfur Batteries. *Nanomaterials* **8**, 90 (2018).
